# *Citrus aurantium* L. Dry Extracts Ameliorate Adipocyte Differentiation of 3T3-L1 Cells Exposed to TNFα by Down-Regulating *miR-155* Expression

**DOI:** 10.3390/nu12061587

**Published:** 2020-05-28

**Authors:** Michele Campitelli, Antonella Desiderio, Giuseppe Cacace, Cecilia Nigro, Immacolata Prevenzano, Alessia Leone, Sonia de Simone, Pietro Campiglia, Pietro Formisano, Gregory A. Raciti, Francesco Beguinot, Claudia Miele

**Affiliations:** 1 URT Genomics of Diabetes, Institute of Experimental Endocrinology and Oncology, National Research Council, 80131 Naples, Italy; mi.campitelli@gmail.com (M.C.); antonella.desid@gmail.com (A.D.); giuseppe.cacace89@gmail.com (G.C.); cecilia.nigro@alice.it (C.N.); imma.prevenzano@libero.it (I.P.); aleleone86@libero.it (A.L.); sonia.des5309@gmail.com (S.d.S.); fpietro@unina.it (P.F.); beguino@unina.it (F.B.); 2 Department of Translational Medicine, Federico II University of Naples, 80131 Naples, Italy; 3 Department of Pharmacy, University of Salerno, 84084 Fisciano, Italy; pcampiglia@unisa.it; 4 European Biomedical Research Institute of Salerno, 84125 Salerno, Italy

**Keywords:** *Citrus aurantium* L. dry extracts, *miR-155*, tumor necrosis factor-alpha, adipogenesis

## Abstract

*Citrus aurantium* L. dry extracts (*CA*de) improve adipogenesis in vitro. These effects are dependent from an early modulation of CCAAT/enhancer-binding protein beta (*C/Ebpβ*) expression and cyclic Adenosine Monophosphate (cAMP) response element-binding protein (*CREB*) activation. *C/Ebpβ* and *Creb* are also targets of *miR-155*. This study investigated whether *CA*de regulates *miR-155* expression in the early stages of adipogenesis and whether it ameliorates adipocyte differentiation of cells exposed to tumor necrosis factor-alpha (TNFα). Adipogenic stimuli (AS) were performed in 3T3-L1 pre-adipocytes treated with *CA*de, TNFα, or both. Gene and miRNA expression were determined by quantitative real-time PCR. Adipogenesis was evaluated by Oil-Red O staining. *CA*de treatment enhanced AS effects during the early adipogenesis phases by further down-regulating *miR-155* expression and increasing both *C/Ebpβ* and *Creb* mRNA and protein levels. At variance, TNFα inhibited 3T3-L1 adipogenesis and abolished AS effects on *miR-155*, *C/Ebpβ*, and *Creb* expression. However, in cells exposed to TNFα, *CA*de improved adipocyte differentiation and restored the AS effects on miRNA and gene expression at early time points. In conclusion, this study identified *miR-155* down-regulation as part of the mechanism through which *CA*de enhances adipogenesis of pre-adipocytes in vitro. Furthermore, it provides evidence of *CA*de efficacy against TNFα negative effects on adipogenesis.

## 1. Introduction

Adipocyte differentiation is a highly orchestrated physiological process, which involves a large number of molecular events and whose dysregulation is relevant to human disease by contributing metabolic dysfunction in obesity [1,2]. microRNAs (miRNAs) represent a class of small non-coding RNAs (≈22 nt in length) involved in a variety of cellular processes, whose role is to repress target translation and to induce target mRNA degradation [3]. Oskowitz et al. were the first to detail miRNA expression during human multipotent stromal cell differentiation toward adipogenic lineage and to demonstrate that adipogenic differentiation and lipid accumulation of these cells is disrupted by inhibition of miRNA biogenesis [4]. In the decade that followed, several miRNAs have been implicated in adipocyte fate determination and adipocyte formation from precursor cells; some of them appear to enhance, while some others appear to inhibit adipocyte differentiation [4,5,6,7,8,9,10,11,12,13,14,15,16,17,18]. For instance, *miR-27a* and *-27b*, belonging to the *miR-27* gene family, were identified as negative regulators of adipogenesis [7,10,11]. The expression of both is indeed down-regulated during adipogenic differentiation of 3T3-L1 cells, and their over-expression is inhibited in vitro adipocyte formation by reducing the levels of master adipogenic regulators such as peroxisome proliferator-activated receptor gamma (*PPARγ*) and CCAAT/enhancer-binding protein alpha (*C/EBPα*) [7]. *miR-130* suppresses adipogenesis by directly targeting and inhibiting *PPARγ* expression [17], while *miR-375* enhances adipocyte differentiation via suppression of extracellular signal-regulated kinases (ERK 1/2) signaling [18].

*miR-155*, initially described as the B-cell integration (*bic*) cluster gene in chickens [19], is a multifunctional miRNA involved in numerous physiological and pathological processes such as haematopoietic lineage differentiation, immunity, inflammation, cancer, and cardiovascular diseases [20,21]. Its role in the regulation of adipocyte differentiation has also been described [22]. Liu et al. indeed reported that *miR-155* expression is down-regulated at the early stage of 3T3-L1 adipogenesis and that its overexpression suppresses adipocyte differentiation by directly targeting the 3′-untranslated regions (3′-UTRs) of *C/ebpβ* and cyclic Adenosine Monophosphate (cAMP) response element-binding protein (*Creb*) mRNAs [22]. In addition, it has been identified as the most responsive miRNA to inflammatory stimuli [22,23], and its expression is higher in the adipose tissue of obese compared to normal-weight subjects and correlates with both tumor necrosis factor-alpha (TNFα) expression and body mass index [23]. It follows that the modulation of the *miR-155* expression in adipocyte precursor cells may offer new ways to enhance adipose tissue homeostasis during obesity.

Nutraceuticals, which encompass all the substances derived from plants and food sources that provide medical or health benefits, have been described as modulating miRNA expression [24,25,26]. As well as this, nutraceuticals are known to regulate adipocyte cell line activity [27]. In line with this, we have recently demonstrated the nutraceutical properties of *CA*de, a dry extract preparation obtained from *Citrus aurantium* L. (*CA*de) fruit juice, on the regulation of 3T3-L1 cell adipocyte differentiation and function [28]. *CA*de enhances in vitro terminal adipocyte differentiation of 3T3-L1 pre-adipocytes in terms of increased gene expression of *Pparγ*, glucose transporter type 4 (*Glut4*), and fatty acid-binding protein 4 (*Fabp4*), as well as the functional capacity of 3T3-L1 mature adipocytes in terms of improved insulin-induced glucose uptake [28]. Furthermore, *CA*de promotes the early differentiation stage as well, by anticipating the 3T3-L1 cell cycle entry and progression during mitotic clonal expansion and by activating *CREB* and nearly doubling the expression of the transcription factor *C/Ebpβ* a few hours later in the adipogenic induction [28].

In the present study, the hypothesis was that *miR-155* and the regulation of its expression are the missing pieces that link *CA*de to *C/Ebpβ* and *CREB* proteins. Furthermore, we also wondered whether *CA*de might be effective against micro-environmental insults, which affect adipogenesis and *miR-155* expression, such as TNFα.

## 2. Materials and Methods

### 2.1. Citrus aurantium L. Dry Extract (CAde)

*Citrus aurantium* L. (*CA*de) fruit juice dry extracts were from [28]. Lyophilized *CA*de was re-hydrated with distilled H_2_O to a final concentration of 10 mg/mL and used for treatments at the concentration of 100 μg/mL.

### 2.2. Cell Culture, Adipocyte Differentiation, and Treatments

Murine embryonic fibroblast 3T3-L1 cells were purchased from the American Type Culture Collection (ATCC, Manassas, VA, USA). Cells were grown and maintained in culture at 37 °C in a humidified atmosphere of 5% CO_2_ in expansion medium consisting of Dulbecco’s modified Eagle’s medium (DMEM; 4.5 g/L glucose; Lonza, Walkersville, MD, USA), 10% fetal calf serum (FCS; Thermo Fisher Scientific, Waltham, MA, USA), 100 U/mL penicillin (Lonza, Walkersville, MD, USA), and 100 mg/mL streptomycin (Lonza, Walkersville, MD, USA) [29,30]. Adipocyte differentiation of 3T3-L1 cells was induced as follows. Cells were grown to confluence in expansion medium. Two days after reaching 100% confluence (day 0), cells were cultured for 48 h with the adipogenic differentiation medium (AS) consisting of DMEM (4.5 g/L glucose), 10% fetal bovine serum (FBS, Thermo Fisher Scientific, Waltham, MA, USA), 100 U/mL penicillin, and 100 mg/mL streptomycin, supplemented with dexamethasone (1 μm, Sigma Aldrich, St Louis, MO), 3-isobutyl-1-methylxanthine (0.5 mm, Sigma Aldrich, St. Louis, MO, USA), and insulin (1 μg/mL, Sigma Aldrich, St. Louis, MO, USA). Forty-eight hours later, the medium was changed, and cells were maintained in culture in AS supplemented with 1 μg/mL insulin, replaced every 48 h, until day 8 post-induction. Where indicated, cells were differentiated with *CA*de (100 μg/mL), or TNFα (1 ng/mL; EMD Millipore, Burlington, MA) or both. *CA*de was added at day 0 and in every replacement. TNFα was added only at day 0. For short-term experiments, 3T3-L1 cells at day 0 were stimulated for 15, 30, 60, 120, and 240 min in AS or in AS supplemented with *CA*de (100 μg/mL), or TNFα (1 ng/mL) or both.

### 2.3. miRNA Mimic and Inhibitor Transfection in Differentiating 3T3-L1

miRNA mimic and inhibitor transfection were performed according to [31]. Briefly, 100% confluent 3T3-L1 cells (day 2) were transfected with 5 nmol·l^−1^ of miRIDIAN mimic *mmu-miR-155-5p* (C-310430-07-0005, Dharmacon Inc., Lafayette, CO, USA) or 5 nmol·L^−1^ of the miRIDIAN Hairpin Inhibitor *mmu-miR-155-5p* (IH-310430-08-0005, Dharmacon Inc.) using Lipofectamine 3000 Reagent (Thermo Fisher Scientific) according to the manufacturer’s instructions. The non-targeting control oligonucleotide miRIDIAN microRNA Mimic negative control #1 (5 nmol·L^−1^; CN-001000-01-05, Dharmacon Inc.) and the non-targeting control oligonucleotide miRIDIAN microRNA Hairpin Inhibitor negative control #1 (5 nmol·L^−1^; IN-001005-01-05, Dharmacon Inc.) were used as a negative control of miRNA mimic and inhibitor transfection, respectively. Forty-eight hours after the transfection (day 0), adipogenesis was induced into cells and left to differentiate into mature adipocytes for a further 8 days, as described above in this section.

### 2.4. Image Acquisition, Oil-Red O Staining, and Triglyceride (TG) Quantification Assay

Images of 3T3-L1 cells at day 8 post-induction were taken using an Olympus microscope system (Olympus, Center Valley, PA, USA). Microphotographs are shown (×10 magnifications); scale bars, 30 μm. Oil-Red O staining was performed as described in [32]. Briefly, 3T3-L1 cells at day 8 post-induction were fixed and stained with Oil-Red O staining solution (Sigma-Aldrich, St. Louis, MO, USA). Lipid accumulation was then quantified by measuring the optical density of the dissolved Oil-Red O staining at 490 nm by a spectrophotometer. Cellular TG concentration was determined according to [33]. Briefly, 3T3-L1 cells at day 8 post-induction were lysed into PBS 1X by sonication. TG content per sample was measured using a TG assay kit from Sigma-Aldrich. Per sample DNA was also isolated using the AllPrep DNA/RNA/miRNA Universal Kit (Qiagen, Hilden, Germany), and DNA concentration was quantified and used to normalize data. The values were expressed as μg Triglyceride (TG) · μg Deoxyribonucleic acid (DNA)^−1^

### 2.5. Total RNA and miRNA Purification, Reverse Transcription, and Quantitative Real-Time PCR

Total RNA, including miRNA, was isolated from 3T3-L1 cells using AllPrep DNA/RNA/miRNA Universal Kit (Qiagen, Hilden, Germany), according to the manufacturer’s instructions. Total RNA concentration was quantified with a NanoDrop 2000 spectrophotometer (Thermo Fisher Scientific, Waltham, MA, USA). Gene expression was determined as described [34]. Total RNA (1000 ng) was reverse-transcribed using the SuperScript III Reverse Transcriptase (Qiagen, Hilden, Germany). Gene expression was analyzed by quantitative real-time PCR using the iQ SYBR Green Supermix (Bio-Rad Laboratories, Inc., Hercules, CA, USA) and quantified as relative expression units. Cyclophilin was used as a housekeeping gene. Primer sequences used are the following: *C/Ebpβ* Fwd, 5′-cgcccgccgcctttagac-3′; *C/Ebpβ* Rev, 5′-cgctcgtgctcgccaatgg-3′; *Creb* Fwd, 5′-agctgccactcagccgggta-3′; *Creb* Rev, 5′-tggtgctcgtgggtgctgtg-3′; Cyclophilin Fwd, 5′-gcaagcatgtggtctttggg-3′; Cyclophilin Rev, 5′-gggtaaaatgcccgcaagtc-3′. miRNA expression was determined as described in [31]. Total RNA (500 ng) was reverse-transcribed using the miScript II RT Kit (Qiagen, Hilden, Germany). miRNA expression was analyzed by quantitative real time-PCR using the miScript SYBR Green PCR Kit (Qiagen, Hilden, Germany) and quantified as relative expression units. U6 small nuclear RNA (snRNA) was used as housekeeping small RNA. Primer sequences were from Qiagen: Mm_miR-155_1 miScript Primer Assay, MS00001701; Mm_miR-130a_1 miScript Primer Assay MS00001547; Mm_miR-375_2 miScript Primer Assay MS00032774; RNU6B_13 miScript Primer Assay, MS00014000.

### 2.6. Western Blot (WB) Analysis

WB analysis was performed as described in [28]. 3T3-L1 cell lysates were obtained by lysing cells in buffer containing 20 mm Tris-HCl, pH 7.5; 5 mm Ethylenediaminetetraacetic acid (EDTA); 150 mm NaCl; 1% Nonidet P40 (NP40), 10 μm phenylmethylsulfonyl fluoride (PMSF); 5 μg/mL aprotinin; and 5 μg/mL leupeptin. Protein concentration was determined by Coomassie blue protein assay (Bio-Rad Laboratories, Hercules, CA, USA). Equal amounts of proteins lysates were then analyzed by SDS-PAGE, and then electrophoretically transferred to a Polyvinylidene fluoride (PVDF) membrane. Membranes were probed with antibodies to total *CREB* (48H2, #9197; Cell Signaling Technology, Danvers, MA, USA), *C/EBPβ* (C-19, sc-150; Santa Cruz Biotechnology, Dallas, TX, USA), and VINCULIN (7F9, sc-73614; Santa Cruz Biotechnology, Dallas, TX, USA), and successively re-probed with secondary mouse or rabbit antibodies (Bio-Rad Laboratories) before signal detection with Enhanced chemiluminescence (ECL) plus (GE Healthcare, Chicago, IL, USA).

### 2.7. Statistical Procedures

Data are given as mean ± standard error of the mean (SEM). One-way analysis of variance (ANOVA) followed by Bonferroni correction post-hoc test was used for comparisons with three or more groups. Data were analyzed by using the GraphPad Software (version 6.00 for Windows, La Jolla, CA, USA).

## 3. Results

### 3.1. CAde Down-Regulated the Expression of miR-155 and Enhanced C/Ebpβ and Creb Levels during the Early Stage of Adipogenesis in 3T3-L1 Cells

**Figure 1:**
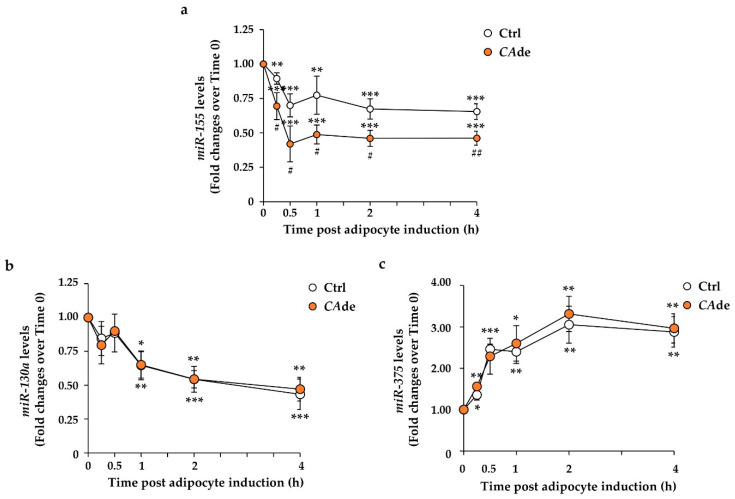
Effect of *Citrus aurantium* L. dry extracts (*CA*de) on the expression of *miR-155* during the early stage of adipogenesis in 3T3-L1 cells. 3T3-L1 pre-adipocytes were cultured for 0.25, 0.5, 1, 2, and 4 h with adipogenic differentiation medium (AS) (Ctrl) or AS + *CA*de (100 μg/mL). Time course of *miR-155* (**a**), *miR-130a* (**b**), and *miR-375* (**c**) levels, evaluated by quantitative real-time PCR, in Ctrl and *CA*de-treated cells relative to untreated 3T3-L1 cells at time 0. Values are means ± Standard Error of the Mean (SEM) of three independent experiments. Control value at time 0 was set as 1.00. Statistical significances among groups were tested by one-way ANOVA followed by Bonferroni correction post-hoc test (* *p* < 0.05, ** *p* < 0.01, and *** *p* < 0.001 vs. untreated 3T3-L1 cells at time 0; ^#^ *p* < 0.05, and ^##^ *p* < 0.01 vs. control 3T3-L1 cells).

To test the hypothesis that *CA*de may potentially exert its pro-adipogenic effects in vitro by modulating *miR-155* expression, the early stage of fat cell differentiation was investigated in 3T3-L1 pre-adipocytes. In control cells, AS induced at 15 min a 10% reduction of the *miR-155* expression, whose levels remained steadily lowered to about 30%–35% at 30 min, 1, 2, and 4 h upon the adipogenic induction (Figure 1a). In AS + *CA*de-treated cells, the expression of *miR-155* resulted in levels being decreased by 31% already by 15 min, and its levels further declined by 58% at 30 min, 51% at 1 h, 54% at 2 h, and 54% at 4 h upon adipogenic induction compared to control cells at time 0 (Figure 1a). Interestingly, compared to AS-treated cells, *CA*de treatment enhanced the effect of AS on *miR-155* down-regulation of by 25%–40% at each time point (Figure 1a and Figure S1a). Additionally, we evaluated *CA*de effect on *miR-130a* and *miR-375*, whose role in adipocyte fate determination has already been demonstrated [17,18]. In control 3T3-L1 cells, AS caused a time-dependent down-regulation of the anti-adipogenic *miR-130a* (Figure 1b) and up-regulation of the pro-adipogenic *miR-375* (Figure 1c) compared to control cells at time 0. Of note, *CA*de treatment did not affect the expression of these two miRNAs. Indeed, in AS + *CA*de-treated cells, the expression levels of both *miR-130a* (Figure 1b and Figure S1b) and *miR-375* (Figure 1c and Figure S1c) at each time point were comparable to those levels observed in AS-treated control cells. Altogether, these findings suggest that *CA*de specifically modulated the expression of *miR-155* in the early stage of fat cell differentiation.

**Figure 2:**
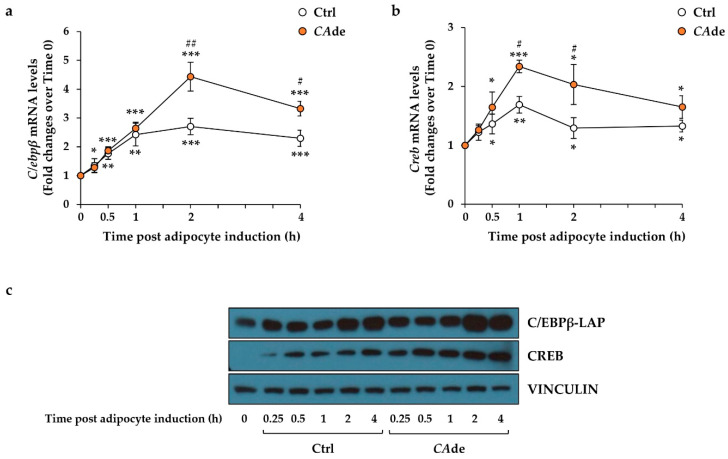
Effect of *CA*de on the expression of the *miR-155* target genes, CCAAT/enhancer-binding protein beta (*C/Ebpβ*) and cyclic Adenosine Monophosphate (cAMP) response element-binding protein (*Creb*), during the early stage of adipogenesis in 3T3-L1 cells. 3T3-L1 pre-adipocytes were cultured for 0.25, 0.5, 1, 2, and 4 h with AS (Ctrl) or AS + *CA*de (100 μg/mL). The *C/Ebpβ* and *Creb* levels were evaluated by quantitative real-time PCR. Time course of *C/Ebpβ* (**a**) and *Creb* (**b**) levels in Ctrl and *CA*de-treated cells relative to untreated 3T3-L1 cells at time 0. Values are means ± SEM of three independent experiments. Control value at time 0 was set as 1.00. Statistical significances among groups were tested by one-way ANOVA followed by Bonferroni correction post-hoc test (* *p* < 0.05, ** *p* < 0.01, and *** *p* < 0.001 vs. untreated 3T3-L1 cells at time 0; ^#^ *p* < 0.05 and ^##^ *p* < 0.01 vs. control 3T3-L1 cells). (**c**) Representative Western blots of the total form of the *C/Ebpβ*-liver-enriched activating protein (LAP), *CREB*, and VINCULIN proteins in control cells and *CA*de-treated cells at 0.25, 0.5, 1, 2, and 4 h after adipogenic induction. C/Ebpβ-LAP and *CREB* protein expression levels at the basal (0) are also reported.

We thus investigated whether *CA*de may consequently affect the expression of the *miR-155* target genes—*C/Ebpβ* and *Creb* [21]. In the control cells, AS led to a time-dependent up-regulation of both *C/Ebpβ* (Figure 2a) and *Creb* (Figure 2b) mRNA levels compared to control cells at time 0. It is worth noting that, compared to AS-treated cells, *CA*de treatment furtherly up-regulated the expression of *C/Ebpβ* by about 65% and 45% at 2 and 4 h, respectively (Figure 2a and Figure S2a), while the combined stimulation of AS and *CA*de up-regulated *Creb* mRNA levels by about 40%, 55%, and 25% at 1, 2, and 4 h, respectively, as compared to treated control cells (Figure 2b and Figure S2b). Coherently with the gene expression data, the protein levels of *C/EBPβ* isoform liver-enriched activating protein (LAP) resulted as being up-regulated at 2 and 4 h from AS in *CA*de-treated cells compared to control cells (Figure 2c). Additionally, the protein levels of *CREB* were increased, starting from 0.25 h from AS in *CA*de-treated cells (Figure 2c). Altogether, these findings at the early stage of fat cell differentiation indicated that *CA*de may exert its function on the adipogenic induction in 3T3-L1 cells, at least in part, by specifically lowering the expression of *miR-155* at the early time points and thus up-regulating the mRNA and protein expression of its target genes *C/Ebpβ* and *Creb*.

### 3.2. CAde Improved Terminal Adipocyte Differentiation of Both 3T3-L1 Cells Over-Expressing miR-155 by Mimic Transfection and 3T3-L1 Cells Exposed to TNFα

Over-expression of *miR-155*, by a gain of function approach [22] or by pro-inflammatory cytokine induction [22,23], has been reported to impair in vitro adipocyte differentiation of 3T3-L1 cells. We then hypothesized that *CA*de might preserve adipogenesis of 3T3-L1 cells, where *miR-155* was over-expressed by mimic transfection or induced by the pro-inflammatory cytokine TNFα.

#### 3.2.1. *miR-155* Gain and Loss of Function Studies

**Figure 3:**
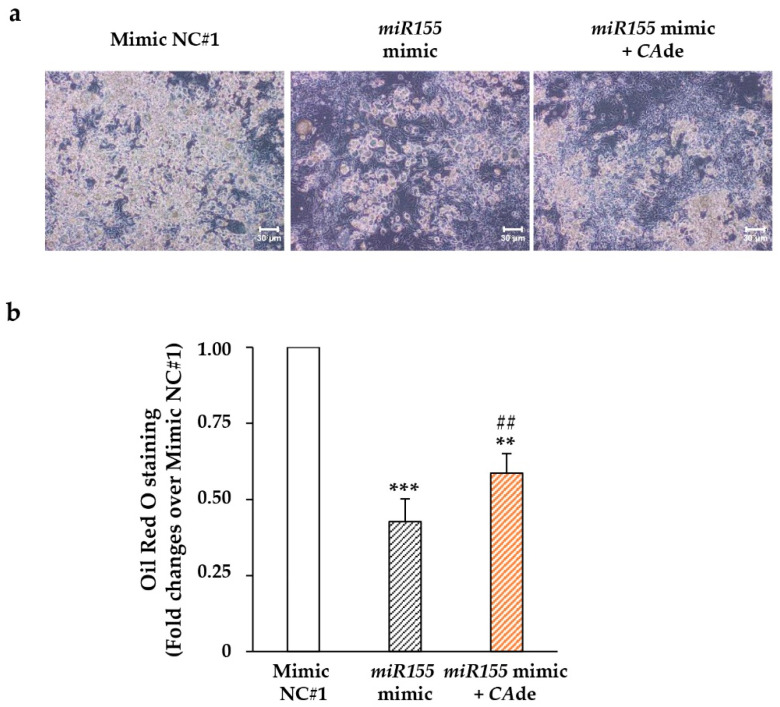
Effect of *CA*de on adipocyte differentiation and lipid accumulation of 3T3-L1 adipocytes over-overexpressing *miR-155*. 3T3-L1 pre-adipocytes were transfected with the mimic negative control (NC) #1 or with the *miR-155* mimic, and were cultured to reach adipocyte differentiation for 8 days with AS. Cells transfected with the *miR-155* mimic were also differentiated with AS supplemented with *CA*de (100 μg/mL). (**a**) Representative microphotographs of adipocytes transfected with mimic negative control (NC) #1 or with the *miR-155* mimic ± *CA*de are shown (X10 magnifications); scale bars, 30 μm. (**b**) Oil-Red O staining of adipocytes transfected with mimic negative control (NC) #1, or with the *miR-155* mimic ± *CA*de. Values are mean ± SEM of determinations from three independent experiments. Statistical significances among groups were tested by one-way ANOVA followed by Bonferroni correction post-hoc test (** *p* < 0.01, and *** *p* < 0.001 vs. mimic negative control (NC) #1; ^##^ *p* < 0.01, *miR-155* mimic + *CA*de vs. *miR-155* mimic).

Fat cell differentiation of 3T3-L1 cells was firstly investigated in cells transfected with the *miR-155* mimic or the *miR-155* inhibitor. The specific over-overexpression of mimic *miR-155* reduced the number of 3T3-L1 cells able to differentiate into adipocytes by about 50%, as shown by light microscopy images (Figure 3a) and Oil-Red O lipid accumulation (Figure 3b). In cells treated with *CA*de, the intracellular lipid accumulation was increased by about 1.4-fold compared to *miR-155* over-overexpressing cells (Figure 3a,b). On the other hand, the specific loss of function of *miR-155* by hairpin inhibitor transfection increased adipogenesis of 3T3-L1 cells by about 90% (Figure S3); no further increase of adipogenesis was observed in 3T3-L1 cells transfected with *miR-155* hairpin inhibitor upon *CA*de treatment (Figure S3). These findings indicated that *CA*de treatment partially prevents the inhibitory effects of *miR-155* on adipocyte differentiation of 3T3-L1 cells.

#### 3.2.2. Treatment with TNFα

**Figure 4:**
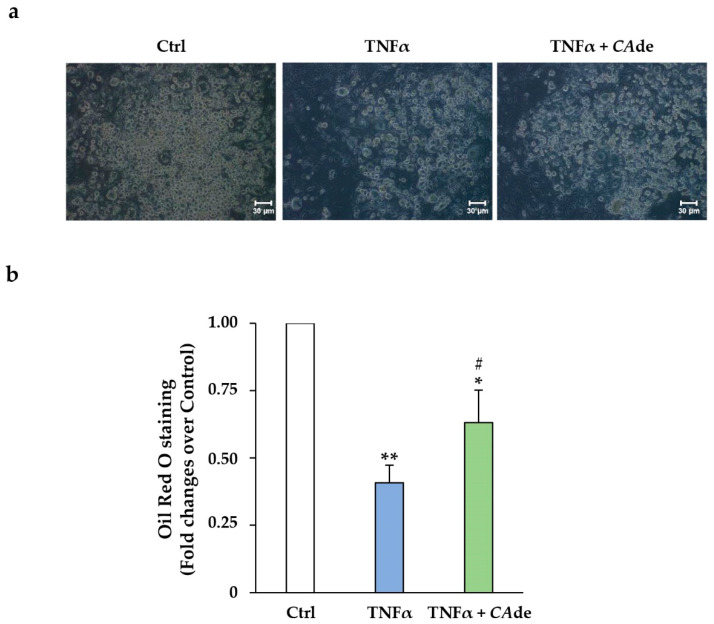
Effect of *CA*de on adipocyte differentiation and lipid accumulation of 3T3-L1 adipocytes treated with tumor necrosis factor-alpha (TNFα). 3T3-L1 pre-adipocytes were cultured to reach adipocyte differentiation for 8 days with AS (Ctrl) or AS supplemented with TNFα (1 ng/mL) ± *CA*de (100 μg/mL). (**a**) Representative microphotographs of Ctrl, TNFα-, and TNFα + *CA*de-treated adipocytes are shown (X10 magnifications); scale bars, 30 μm. (**b**) Oil-Red O staining of Ctrl, TNFα-, and TNFα + *CA*de-treated adipocytes. Values are mean ± SEM of determinations from three independent experiments. Statistical significances among groups were tested by one-way ANOVA followed by Bonferroni correction post-hoc test. (* *p* < 0.05, and ** *p* < 0.01 vs. Ctrl; ^#^ *p* < 0.05, TNFα + *CA*de vs. TNFα).

Secondly, we investigated whether *CA*de may preserve adipogenesis of cells exposed to the pro-inflammatory cytokine TNFα, which exerts profound inhibition of adipocyte differentiation by *miR-155* induction [21,22]. As expected, the number of 3T3-L1 pre-adipocytes able to achieve full differentiation was strongly reduced by TNFα treatment, as shown by the light microscopy images (Figure 4a). Consistent with this, TNFα reduced the intracellular lipid accumulation (Figure 4b) by about 60% and the TG deposition by about 70% (Ctrl, 41.5 ± 0.3; TNFα, 11.2 ± 0.3, μg TG · μg DNA^−1^; *p* < 0.001) compared to control adipocytes. It is worth noting that in cells co-treated with TNFα and *CA*de, the number of adipocytes was increased compared to TNFα-treated cells (Figure 4a). In addition, upon *CA*de treatment, the intracellular lipid accumulation (Figure 4b) and TG deposition were increased by 1.5- and about 2.2-fold, respectively, compared to TNFα-treated adipocytes (TNFα + *CA*de, 26.4 ± 5.8; TNFα, 11.2 ± 0.3, μg TG · μg DNA^−1^; *p* < 0.05). Altogether, these data suggest that *CA*de treatment partially protected the adipogenesis of 3T3-L1 cells from the TNFα inhibitory effect.

### 3.3. CAde Prevented TNFα-Induced Dysregulation of miR-155, C/Ebpβ, and Creb Expression during the Early Stage of Adipogenesis in 3T3-L1 Cells

**Figure 5:**
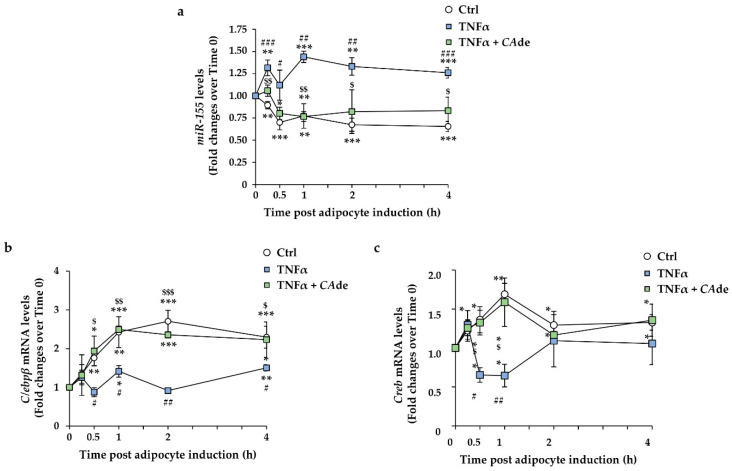
Effect of *CA*de on the expression of *miR-155* and its target genes, *C/Ebpβ* and *Creb*, during the early stage of adipogenesis in 3T3-L1 cells exposed to TNFα. 3T3-L1 pre-adipocytes were cultured for 0.25, 0.5, 1, 2, and 4 h with AS (Ctrl) or AS supplemented with TNFα (1 ng/mL) ± *CA*de (100 μg/mL). The miRNA and gene expressions were evaluated by quantitative real-time PCR. Time course of *miR-155* (**a**), *C/Ebpβ* (**b**), and *Creb* (**c**) levels in Ctrl, TNFα-, and TNFα + *CA*de-treated cells are shown relative to untreated 3T3-L1 cells at time 0. Values are means ± SEM of three independent experiments. Control value at time 0 was set as 1.00. Statistical significances among groups were tested by one-way ANOVA followed by Bonferroni correction post-hoc test (* *p* < 0.05, ** *p* < 0.01, and *** *p* < 0.001 vs. untreated 3T3-L1 cells at time 0; ^#^ *p* < 0.05, ^##^ *p* < 0.01 and ^###^ *p* < 0.001 vs. control 3T3-L1 cells; ^$^ *p* < 0.05, ^$$^ *p* < 0.01, ^$$$^ *p* < 0.001 vs. TNFα-treated 3T3-L1 cells).

Up-regulation of *miR-155* and suppression of *C/Ebpβ* and *Creb* expression were identified as one of the mediators of the TNFα-dependent inhibition of adipogenesis [22,23]. We thus evaluated whether *CA*de may counteract these anti-adipogenic effects of TNFα during the early time points upon adipogenic induction of 3T3-L1 pre-adipocytes. As expected, TNFα already at 15 min from adipogenic induction induced a 30% increase in *miR-155* expression, whose level remained elevated at 30 min, 1, 2, and 4 h upon AS stimulation compared to control 3T3-L1 cells at time 0 (Figure 5a). Consistent with this, TNFα treatment also decreased the expression of *C/Ebpβ* by about 50% at 30 min, 42% at 1 h, 66% at 2 h, and 35% at 4 h upon AS stimulation (Figure 5b) and by *Creb* by about 52% at 30 min and 62% at 1 h upon AS stimulation (Figure 5c). Interestingly, the co-treatment with TNFα and *CA*de down-regulated *miR-155* expression during the early time points of adipogenic induction to levels comparable to AS-treated control cells (Figure 5a and Figure S4a). At the same time points upon adipogenic induction, an up-regulation of both *C/Ebpβ* (Figure 5b and Figure S4b) and *Creb* (Figure 5c and Figure S4c) mRNA expression, to levels comparable to those in AS-treated control cells, was observed in the TNFα + *CA*de-treated cells. Altogether, these findings suggest that *CA*de weakened the inhibitory effect of TNFα on the adipogenesis of 3T3-L1 cells by restoring the AS effects on *miR-155* down-regulation and *C/Ebpβ* and *Creb* gene up-regulation at the early differentiation time points.

## 4. Discussion

miRNAs have emerged as critical regulators of a variety of biological processes in eukaryotic cells, and the deregulation of their function is associated with an increasing number of human diseases [3]. Furthermore, it has been reported that miRNAs may contribute to the metabolic abnormalities associated with obesity and obesity-related complications by particularly affecting the function of the white adipose tissue (WAT) [35,36]. Indeed, in human WAT, numerous miRNAs are expressed, obesity influences their expression, and their predominant function is to stimulate or inhibit the differentiation of pre-adipocytes into adipocytes, as well as to regulate specific metabolic and endocrine functions, as revealed through loss or gain of function studies on the obesity-associated miRNAs [35,36,37,38,39,40,41]. This makes miRNAs a tangible target for the treatment of adipocyte dysfunction and its related disorders.

Nutraceuticals have been used for decades now in weight management in obese individuals and are described to modulate adipogenesis and to have other positive effects on obesity pathogenesis [27]. Growing evidence sustains the hypothesis that dietary modulation of miRNA expression may explain in part some of the beneficial effects of nutraceuticals on health [24,25,26]. Indeed, an increasing number of studies have reported that several natural food-derived compounds modulate miRNA expression in different animal cells and tissues [24,25,26]. Extracts from *Citrus aurantium* L. have been recently used for investigating adipocyte differentiation [28,42,43]. However, quite different results have been obtained, and whether or not extracts from *Citrus aurantium* L. have pro- or anti-adipogenic properties is still unclear. Kim et al. have indeed reported on the anti-adipogenic effect of preparation from the fruit peel of *Citrus aurantium* L. in 3T3-L1 cells, where the flavonoids naringin, hesperidin, and nobiletin are predominant [42]. Park et al. have instead demonstrated in 3T3-L1 cells and primary cultured adipocytes the anti-adipogenic and pro-thermogenic actions, respectively, of preparation from the immature dried fruit of *Citrus aurantium* L., which is abundant in naringin and neohesperidin [43]. We have recently shown that *CA*de, a dry extract preparation obtained from the fruit juice of *Citrus aurantium* L., mainly enriched in hesperidin, narirutin, and vicenin-2, increased adipocyte differentiation and function of 3T3-L1 cells [28]. Therefore, reasons for these controversial findings could probably be found on the specificity and the amounts of flavonoids within each of these *Citrus aurantium* L. extracts, which also depends on their origin.

In the present study, we have further reported new evidence that sustains nutraceutical beneficial effects of *CA*de on the regulation of miRNA expression and function in vitro in 3T3-L1 pre-adipocytes. In particular, we demonstrated that the treatment of cells with *CA*de enhanced the down-regulation of the adipogenic suppressor *miR-155* [22,23], as early as 15 min upon induction of adipogenesis in 3T3-L1 pre-adipocytes. This results in an up-regulation of the mRNA and protein levels of the *miR-155* target genes, *C/Ebpβ* and *Creb* [22]. Specifically, *CA*de treatment further increased the expression of *C/Ebpβ* mRNA and of the pro-adipogenic *C/EBPβ*-LAP protein isoform upon 2 h from the adipogenic induction [44]. Additionally, *CA*de further up-regulated *Creb* mRNA and protein levels.

It is worth noting that *CA*de specifically modulated *miR-155* expression during the early stage of adipogenesis. Indeed, *CA*de treatment during the first 4 h post-adipogenic induction with AS did not affect the expression of other miRNAs, such as *miR-130a* and *miR-375*, whose role in adipocyte fate determination has been already demonstrated [17,18,41]. *miR-155* is biologically relevant to the regulation of adipocyte differentiation, and its perturbation is associated with obesity [22,23]. In addition, *miR-155* expression in cells is modulated by nutraceutical compounds [45,46,47]. Indeed, Eseberri et al. have shown that the stilbenoid resveratrol and its metabolites, trans-resveratrol-3-*O*-glucuronide and trans-resveratrol-4-*O*-glucuronide, exert their anti-adipogenic effect on 3T3-L1 cells by up-regulating *miR-155* expression [45]. On the contrary, others reported the down-regulation of the same miRNA by flavonoid treatment [46,47]. Boesch-Saadatmandi et al. have indeed demonstrated that the flavonoid quercetin and its metabolite isorhamnetin partially neutralize lipopolysaccharide-induced increase of *miR-155* in murine RAW264.7 macrophages and state that *miR-155* inhibition possibly contributes to the anti-inflammatory properties of both flavonoids [46]. Additionally, Arango et al. have reported that the flavonoid apigenin and a celery-based apigenin-rich diet exert effective anti-inflammatory activity in vivo by reducing expression of *miR-155* [47]. As reported above in this section, flavonoids, such as hesperidin, narirutin, and vicenin-2, are very abundant in our dry extract preparation [28] and their presence might be thus responsible for the observed down-regulation of *miR-155* expression in *CA*de-treated cells.

Here, we have also reported that *CA*de partially preserved adipogenesis of 3T3-L1 cells, where the expression of *miR-155* was up-regulated by mimic transfection. *CA*de did not further enhance adipogenesis of 3T3-L1 cells, where *miR-155* activity was abolished by specific hairpin inhibitor. These findings led us to suppose that the treatment with *CA*de may be effective against any micro-environmental insults, which impair adipocyte differentiation by up-regulation of *miR-155*. In accordance with this, we indeed found that the *CA*de treatment counteracted the detrimental effects of TNFα on adipogenesis. Indeed, terminal adipocyte differentiation of 3T3-L1 cells exposed to TNFα was improved by almost 50% by *CA*de and was associated with a restoring of the expression of *miR-155*, *C/Ebpβ*, and *Creb* during the early stage of adipogenesis. TNFα is a pleiotropic cytokine that exerts homeostatic and pathogenic bioactivities [48]. High TNFα levels are observed in the WAT during obesity, and they have profound effects on adipocyte metabolism by impairing triglyceride synthesis and storage and inhibiting adipocyte differentiation [22]. Liu et al. have also demonstrated in 3T3-L1 pre-adipocytes that *miR-155*, whose expression is up-regulated by TNFα as early as 5 min via NFκB-p65 (nuclear factor kappa-light-chain-enhancer of activated B cells) binding to the *miR-155* promoter, mediates at least in part the TNFα-induced suppression of adipogenesis by down-regulating early adipogenic transcription factors [22]. These findings, therefore, provide the first piece of evidence for the efficacy of *CA*de treatment in vitro against micro-environment insults deleterious for the functional capacity of adipose cells. In this scenario, *CA*de may ameliorate, in the early stage, the differentiation process by blocking NFκB-p65 into the cytosol and thus preventing the NFkB-p65–mediated transcription of *miR-155*. This hypothesis was indeed sustained by our preliminary data in 3T3-L1 pre-adipocytes short-term treated with TNFα, where the TNFα-induced NFκB-p65 translocation from cytosol to the nucleus was prevented by *CA*de treatment.

In conclusion, this study demonstrated that *miR-155* down-regulation is part of the mechanism through which *CA*de enhances adipocyte differentiation of pre-adipocytes in vitro. In addition, we herein provide substantial evidence of the efficacy of this nutraceutical compound against micro-environment insults, which are harmful to adipose cell functionality and affect *miR-155* expression, such as TNFα, suggesting that the development of *CA*de-derived compounds may be an effective strategy for the treatment of adipocyte dysfunction and its related disorders.

## Supplementary Materials

The following are available online at https://www.mdpi.com/2072-6643/12/6/1587/s1.
