# Supplementary material for: Citrus aurantium L. Dry Extracts Ameliorate Adipocyte Differentiation of 3T3-L1 Cells Exposed to TNFα by Down-Regulating miR-155 Expression
Source: Nutrients. 2020 May 28;12(6):1587. doi: 10.3390/nu12061587 (PMC7352926; doi:10.3390/nu12061587)
Supplement: Supplementary file 1 [file nutrients-12-01587-s001.zip › Figure S legends.pdf]

Figure S legends

Figure S1. Expression of *miR-155*, *miR-130a* and *miR-375* during the early stage of adipogenesis in 3T3-L1 cells un-treated or treated with *CA*de. 3T3-L1 pre-adipocytes were cultured for 0.25, 0.5, 1, 2 and 4 h with AS (Ctrl) or AS + *CA*de (100 μg/ml). The levels of *miR-155*, *miR-130a* and *miR-375* were evaluated by quantitative real-time PCR. *miR-155* (**a**), *miR-130a* (**b**) *and miR-375*(**c**) expression in *CA*de-treated cells at 0.25, 0.5, 1, 2 and 4 h post adipogenic induction relative to their levels in un-treated control cells at the same time points. Values are means ± SEM of three independent experiments. Control values at 0.25, 0.5, 1, 2 and 4 h post adipogenic induction were set as 1.00. Statistical significances among groups were tested by one-way ANOVA followed by Bonferroni correction post hoc test. $^{\#}p<0.05$, and $^{\#\#}p<0.01$ vs control 3T3-L1 cells.

Figure S2. Expression of the *miR-155* target genes, *C/Ebpβ* and *Creb*, during the early stage of adipogenesis in 3T3-L1 cells un-treated or treated with *CA*de. 3T3-L1 pre-adipocytes were cultured for 0.25, 0.5, 1, 2 and 4 h with AS (Ctrl) or AS + *CA*de (100 μg/ml). The *C/Ebpβ* and *Creb* levels were evaluated by quantitative real-time PCR. The levels of *C/Ebpβ* and *Creb* were evaluated by quantitative real-time PCR. *C/Ebpβ* (**a**) and *Creb* (**b**) expression in *CA*de-treated cells at 0.25, 0.5, 1, 2 and 4 h post adipogenic induction relative to their levels in un-treated control cells at the same time points. Values are means ± SEM of three independent experiments. Control values at 0.25, 0.5, 1, 2 and 4 h post adipogenic induction were set as 1.00. Statistical significances among groups were tested by one-way ANOVA followed by Bonferroni. $^{\#}p<0.05$, and $^{\#\#}p<0.01$ vs control 3T3-L1 cells.

Figure S3. Adipocyte differentiation and lipid accumulation of 3T3-L1 adipocytes silenced for *miR-155*. 3T3-L1 pre-adipocytes were transfected with hairpin inhibitor negative control (NC) #1, or with *miR-155* hairpin inhibitor and were cultured to reach adipocyte differentiation for eight days with AS. Cells transfected with the *miR-155* hairpin inhibitor were also differentiated with AS supplemented with *CA*de (100 μg/ml). (**a**) Representative microphotographs of adipocytes transfected with hairpin inhibitor negative control (NC) #1, or with *miR-155* hairpin inhibitor ± *CA*de are shown (X10 magnifications); scale bars, 30 μm. (**b**) Oil Red O staining of adipocytes transfected with hairpin inhibitor negative control (NC) #1, or with *miR-155* hairpin inhibitor ± *CA*de. Values are mean ± SEM of determinations from three independent experiments. Statistical significances among groups were tested by one-way ANOVA followed by Bonferroni correction post hoc test (**$p<0.01$, and ***$p<0.001$ vs hairpin inhibitor negative control (NC) #1).

Figure S4. Expression of *miR-155*, *C/Ebpβ* and *Creb* during the early stage of adipogenesis in 3T3-L1 cells treated with TNF*α* or with TNF*α* and *CA*de. 3T3-L1 pre-adipocytes were cultured for 0.25, 0.5, 1, 2 and 4 h with AS (Ctrl) or AS supplemented with TNF*α* (1 ng/ml) ± *CA*de (100 μg/ml). The miRNA and gene expression were evaluated by quantitative real-time PCR. The levels of *miR-155*, *C/Ebpβ and Creb* were evaluated by quantitative real-time PCR. *miR-155* (**a**), *C/Ebpβ* (**b**) *and Creb* (**c**) expression in *CA*de-treated cells at 0.25, 0.5, 1, 2 and 4 h post adipogenic induction relative to their levels in un-treated control cells at the same time points. Values are means ± SEM of three independent experiments. Control values at 0.25, 0.5, 1, 2 and 4 h post adipogenic induction were set as 1.00. Statistical significances among groups were tested by one-way ANOVA followed by Bonferroni correction post hoc test. ($^{\#}p<0.05$, $^{\#\#}p<0.01$ and $^{\#\#\#}p<0.001$ vs control 3T3-L1 cells; $^{\$}p<0.05$, $^{\$\$}p<0.01$, $^{\$\$\$}p<0.001$ vs TNF*α*-treated 3T3-L1 cells).
