# Supplementary figures and images for: Citrus aurantium L. Dry Extracts Ameliorate Adipocyte Differentiation of 3T3-L1 Cells Exposed to TNFα by Down-Regulating miR-155 Expression
Source: Nutrients. 2020 May 28;12(6):1587. doi: 10.3390/nu12061587 (PMC7352926; doi:10.3390/nu12061587)

**a**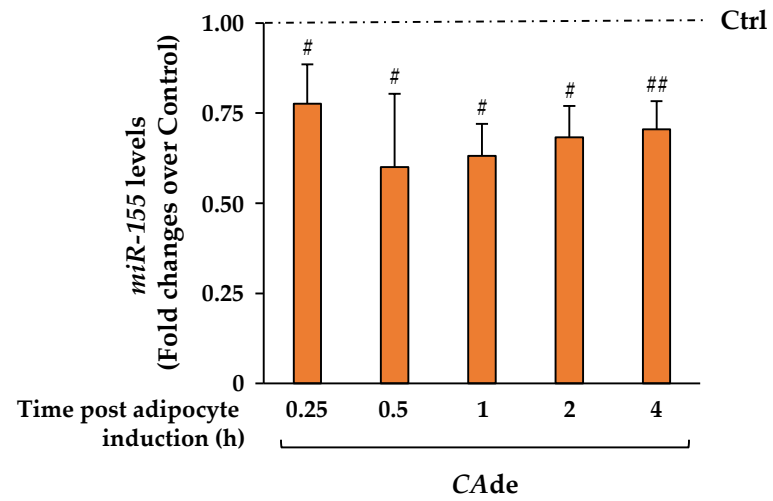**b**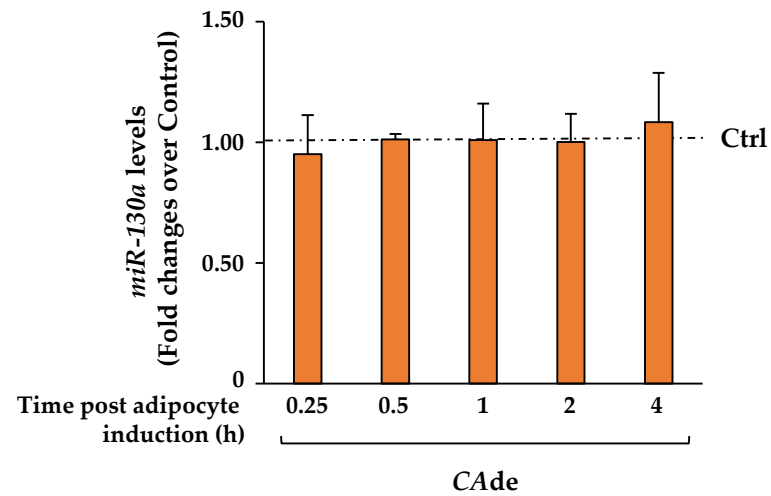**c**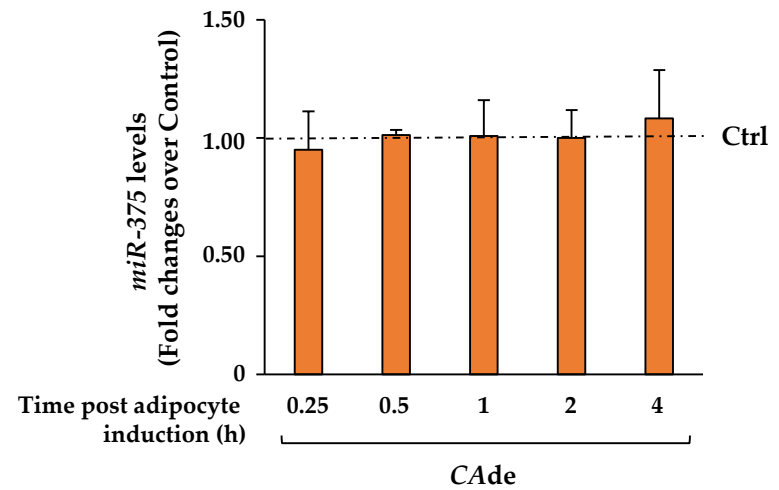**Figure S1**

Supplement: Supplementary file 1 [file nutrients-12-01587-s001.zip › Figure S1.pdf]

**a**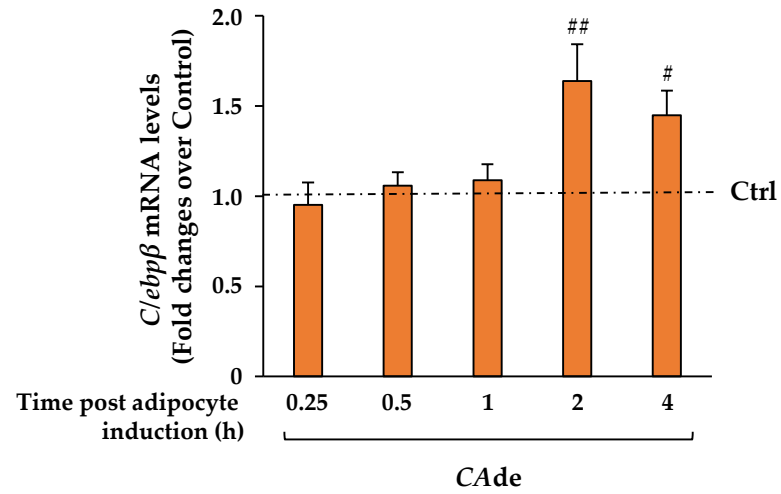**b**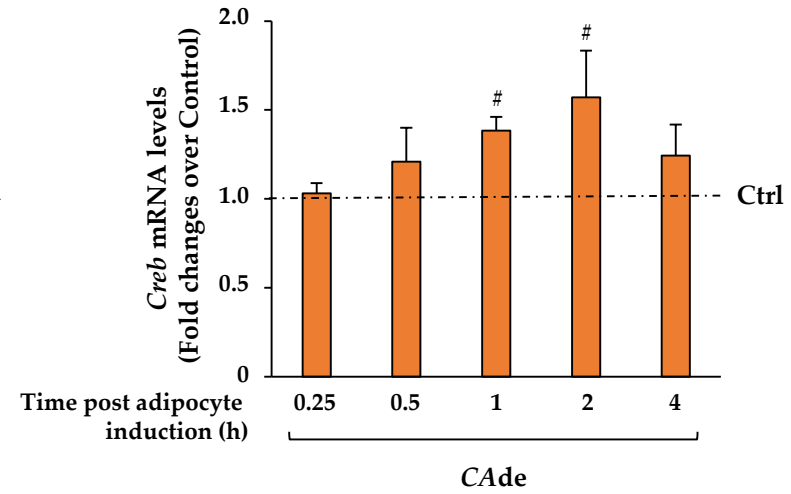**Figure S2**

Supplement: Supplementary file 1 [file nutrients-12-01587-s001.zip › Figure S2.pdf]

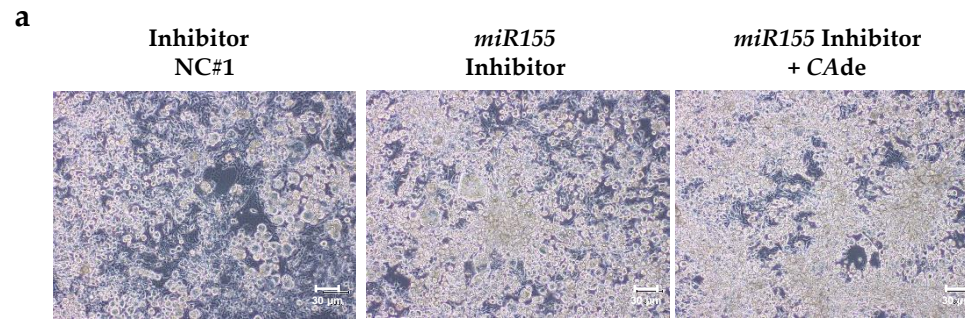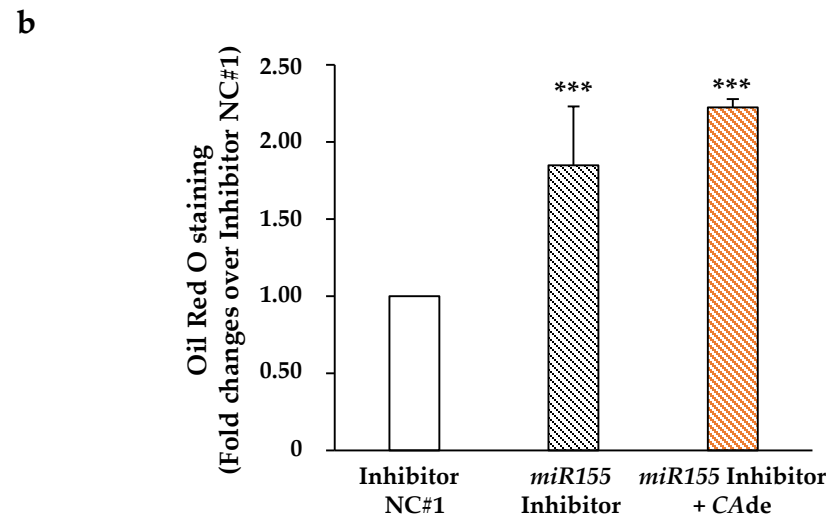

Figure S3

Supplement: Supplementary file 1 [file nutrients-12-01587-s001.zip › Figure S3.pdf]

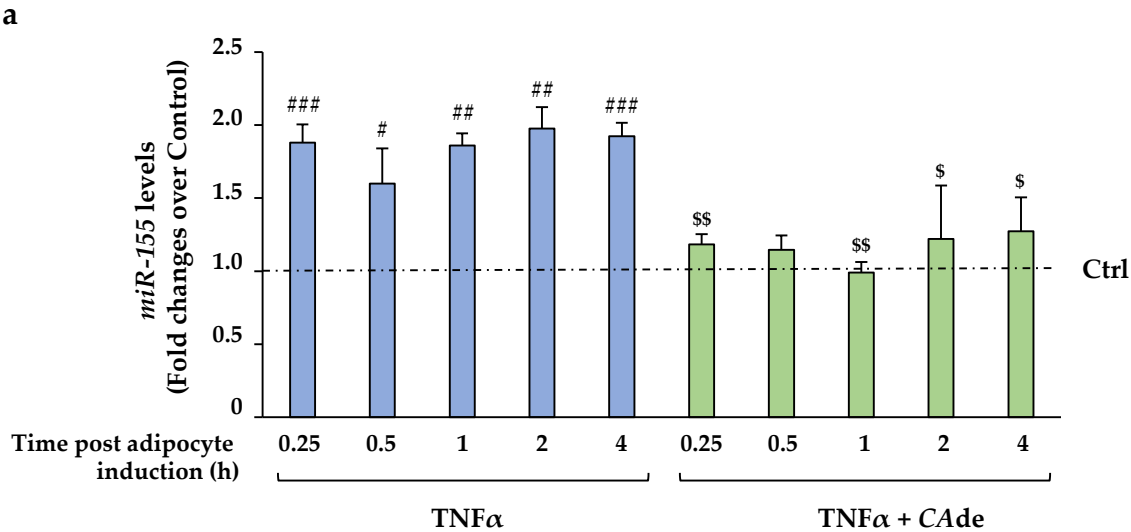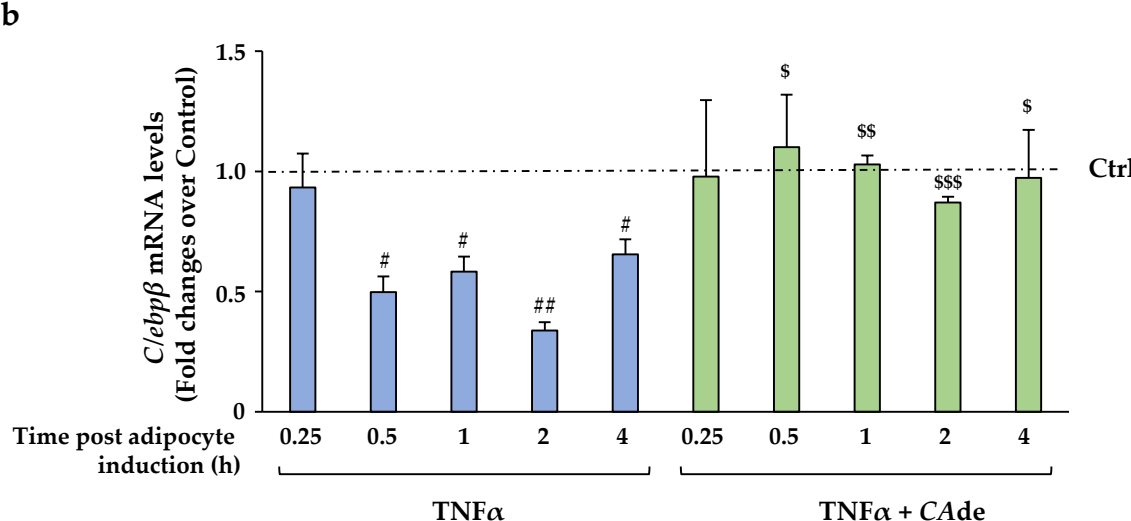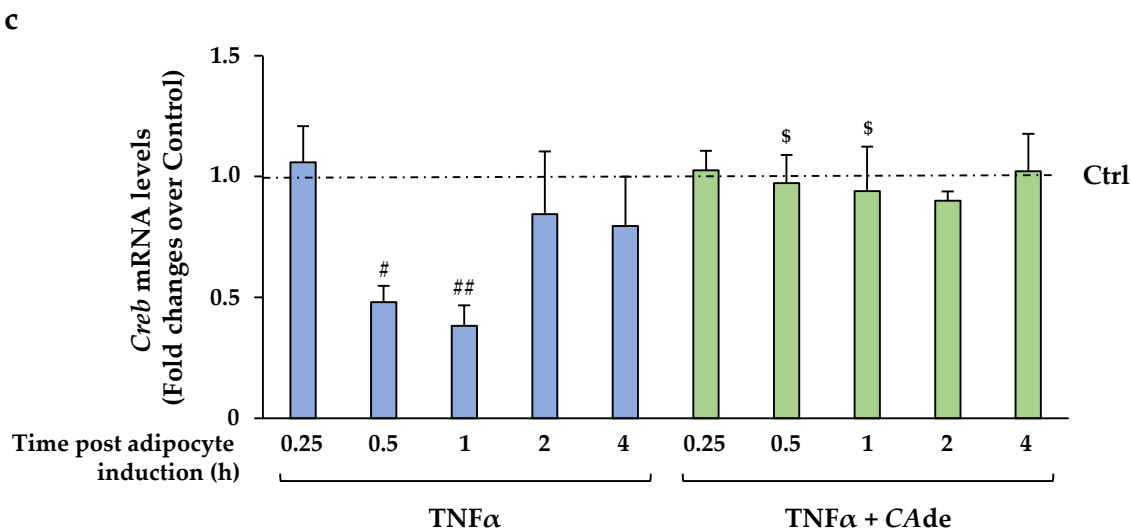

Figure S4

Supplement: Supplementary file 1 [file nutrients-12-01587-s001.zip › Figure S4.pdf]
